# Efficacy and safety of kaolin-based hemostatic pad vs. standard mechanical compression following transradial and transulnar access for elective coronary angiography and PCI: RAUL trial substudy

**DOI:** 10.1007/s00380-019-01520-z

**Published:** 2019-10-26

**Authors:** Lewandowski Pawel, Gralak-Lachowska Dagmara, Maciejewski Pawel, Ramotowski Bogumil, Budaj Andrzej, Stec Sebastian

**Affiliations:** 1 Centre of Postgraduate Medical Education, Grochowski Hospital, Cardiology Department, Grenadierow 51/59, 04-073 Warsaw, Poland; 2 Chair of Electroradiology, Faculty of Medicine, University of Rzeszow, Rzeszow, Poland

**Keywords:** Transradial access, Transulnar access, Hemostasis device

## Abstract

Hemostatic devices used in the transradial approach (TRA) and transulnar approach (TUA) are limited. This study compared the efficacy and safety of hemostasis using the QuikClot Radial hemostatic pad (QC) vs. standard mechanical compression (SC) after coronary angiography (CAG). This prospective single-center randomized trial included CAG patients. The primary and secondary endpoints were efficacy (successful hemostasis) and safety (total artery occlusion [TAO], pseudoaneurysm, hematoma), respectively. A visual analog scale (VAS) evaluated patient pain during compression. In 2013–2017, 200 patients were randomized 2 × 2 into the: (1) TRA and TUA groups and (2) QC and SC groups. Successful hemostasis was achieved in 92 (92%) patients in the QC group and 100 (100%) patients in the SC group (*p* < 0.006). The TRA SC subgroup showed significantly better results than the TRA QC subgroup (100% vs. 90.0%; *p* < 0.03). Similar results were obtained in the TUA QC and TUA SC subgroups (95% vs. 100%; *p* = 0.5). The secondary endpoint was achieved in the QC and SC groups (8% vs. 9%; *p* = 0.8). Patients reported significantly less pain during QC application than during SC (VAS: 2.6 ± 2.6 vs. 3.4 ± 2.9; *p* < 0.03). In patients undergoing CAG with TRA or TUA, QC was associated with lower efficacy, less discomfort, and similar safety compared to SC.

## Introduction

Access-site hemostasis plays a crucial role in successful transradial interventions [1, 2]. Moreover, the use of special hemostatic devices may shorten the hemostasis time and reduce the incidence of vascular site complications, including bleeding, pseudoaneurysms (IPA), and total artery occlusion (TAO), compared to standard manual compression (SC) [3–5]. Many hemostatic devices have been developed with the aim of improving TRA access-site hemostasis and limiting local complications; however, no TUA-dedicated access-site hemostasis device is currently available [3, 4, 6, 7]. The QuikClot Radial™ (QC) (Z-Medica Corporation, Wallingford, CT, USA) is a hemostatic device that is composed of a kaolin-impregnated sterile roll and a direct wound pressure system. Kaolin initiates the clotting cascade by activating factor XII [8, 9]. QC hemostatic pads achieved sooner femoral artery (FA) hemostasis and allowed earlier ambulation and a shorter hospital stay than SC after PCI [10, 11]. A recent study showed a trend toward a decreased incidence of TAO with a QC pad compared to conventional radial artery gauze compression within 24 h and significantly shorter hemostasis time compared to the TR Band™ (Terumo Medical Corporation, Tokyo, Japan) [12, 13].

In our department, an adhesive bandage (Peha-haft, Paul Hertmann AG, Heidneheim, Germany) is used as hemostatic device for TRA and TUA and a ruled bandage swathed with adhesive tape is used as a rectangular hard pad (Fig. 2b).

The aim of this study was to compare the efficacy and safety of QC and SC in patients scheduled for elective CAG with TRA and TUA.

## Methods

### Study design

**Fig. 1:**
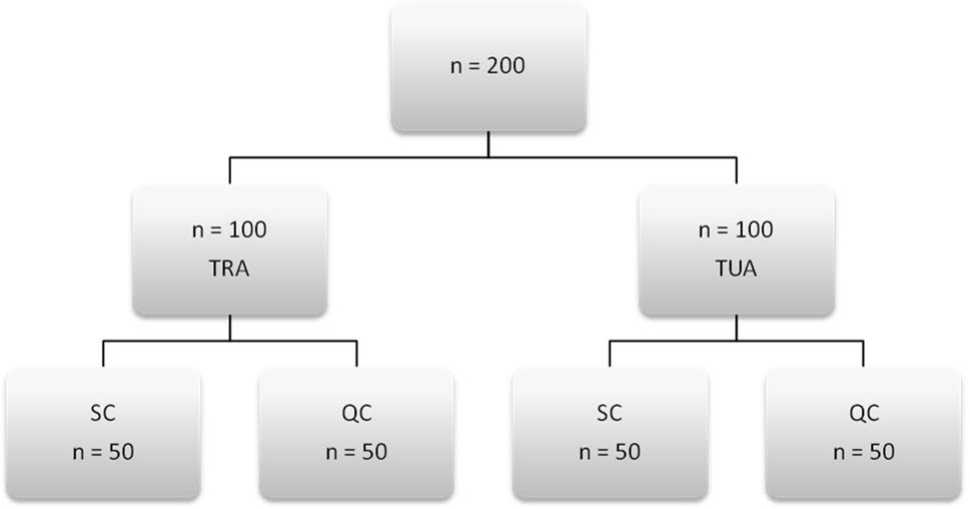
Patient randomization. *QC* QuikClot group, *SC* standard compression group, *TRA* transradial approach, *TUA* transulnar approach

This prospective single-center randomized study was conducted between 2013 and 2017 (TransRadial versus transUlnar artery approach for elective invasive percutaneous coronary interventions: a randomized trial on the feasibility and safety with ultrasonography outcome—RAUL study). A total of 200 patients who were referred for their first elective CAG were included in the study. Eligible patients were then randomly assigned to the TRA or TUA and QC or SC groups. The randomization allocation is presented in Fig. 1. The interventional cardiologists were blinded to the randomization results and ultrasound parameters. Regardless of sex, patients who were at least 18 years of age and hospitalized for their first elective CAG were eligible to participate in the trial. Patients with upper-limb anomalies and those who underwent prior vascular interventions (TRA or TUA) were excluded. The additional exclusion criteria were as follows: RA or UA < 1.5 mm based on preprocedural ultrasonography, and a positive Allen’s test results. Before the invasive procedure, all patients underwent 2-dimensional ultrasonography (US) and Doppler measurements of the upper limb (EUB 5000 ultrasound scanner; Hitachi, Tokyo, Japan; Transducer: linear array, 5–10 MHz). Vessel diameter, distance from the skin to the anterior wall of the artery, peak systolic velocity (PSV) of the blood in the RA, and UA were measured. All parameters of the RA and UA were measured about 2 cm above the wrist at the place of the potential puncture. US was performed before the CAG within the first 24 h after the procedure and then after 1, 3, and 6 months. Right upper-limb access was the first choice. Standard radial introducers (6-Fr; Radial Introducer Sheath, Demax Medical Co. Ltd., Sydney, Australia), 6-Fr or 5-Fr diagnostic catheters (Angiodyn, B Braun, Melsungen, Germany), and 6-Fr guiding catheters (Luncher, Medtronic, Danvers, MA, USA) were used for the CAG and PCI. After vascular access was established, a bolus of unfractionated heparin (UFH) 5000 IU was administered and angiography of the upper limb circulation was performed. At least 0.2 mg of intraarterial nitroglycerine was administered in case of vascular spasm. According to standard protocols, CAG was performed with ad hoc PCI as necessary. Before CAG, the subjects were randomized to one of the two hemostasis protocols: QC with 120 min of compression or SC with 120 min of compression.

### QuikClot Radial™ procedure

**Fig. 2:**
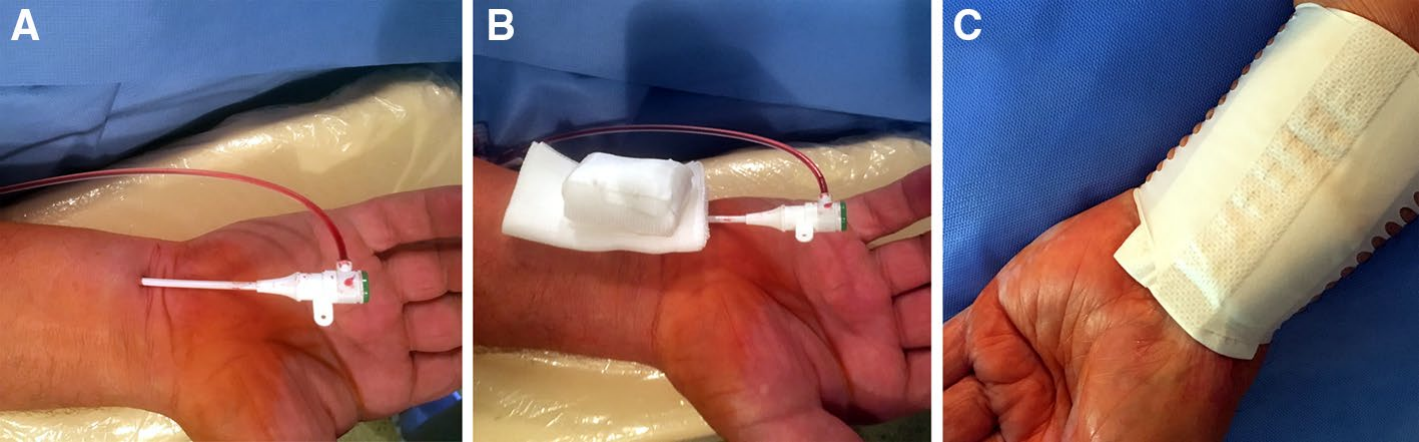
Two methods of hemostasis. **a** Vascular sheath in the ulnar artery (UA). **b** Rectangular shaped hard pad prepared for application (standard compression). **c** Properly applied QuikClot pad over the UA

At the end of the CAG or PCI procedure, the vascular sheath was pulled out and a small amount of blood was allowed to soak the underside of the QC roll to initiate the kaolin–blood reaction. Manual compression was then applied to the QC roll for 5 min to obtain hemostasis. Finally, the elastic bandage system was applied to provide direct wound pressure (Fig. 2c). After 30 min, compression was gradually released by cutting every other elastic band on both sides. After 120 min, the hemostatic system was completely removed regardless of vascular access (TRA or TUA; Fig. 2a). Sterile gauze with adhesive tape was then applied to the puncture site. If hemostasis failed, SC was applied for an additional 120 min.

### SC procedure

After vascular sheath removal, sterile gauze was positioned on the skin over the wound and a roll and cohesive bandage were applied. The maximum hemostatic time for TRA was 120 min. After 30 min, one-third of the cohesive bandage was removed to improve the blood circulation and limit patient complaints. After 2 h, the hemostatic system was removed. Sterile gauze with adhesive tape was then applied to the puncture site (Fig. 2b). If hemostasis failed (during the hemostatic procedure or after SC removal), standard compression was applied for an additional 120 min.

The primary endpoint was efficacy defined as successful hemostasis without bleeding during or after removal hemostatic device with no evidence of an expanding forearm hematoma.

The secondary endpoint was safety assessed by several vascular complications such as TAO, IPA, or large hematoma. Major complications were defined as: major bleeding (type III and V as per the Bleeding Academic Research Consortium) [14], large hematoma of the forearm (4 grade in EASY scale), IPA, and TAO confirmed by US examination. Forearm hematomas were classified into four grades based on size: grade 1, less than 5 cm in size; grade 2, less than 10 cm; grade 3, extended distal to the elbow; and grade 4, extended above the elbow (EASY scale) [15]. VAS was obtained 1 h after compression device removal. A visual analog scale (VAS) was used to evaluate patient pain associated with both compression methods [16].

### Statistical analysis

Quantitative variables are expressed as mean (± standard deviation) or median and interquartile range if a deviation from the normal assumption was observed using the Shapiro–Wilk test. Differences in these variables were compared using Student’s *t* test or the Mann–Whitney *U* test as appropriate. Qualitative variables are summarized by percentage count and percentage and were compared using the chi-squared test or Fisher’s exact test when there were fewer than five expected events. Two-sided *p* values < 0.05 were considered statistically significant. SAS software version 9.2 (SAS Institute, Cary, NC, USA) was used for all the analyses.

## Results

Between 2013 and 2017, 200 patients were randomized in a 2 × 2 fashion to: (1) TRA and TUA groups; and (2) QC and SC groups. The main indication for CAG was suspected coronary artery disease. Statistically significant differences in age were found between the QC and SC groups. The baseline characteristics of the included patients are summarized in Table 1. Data on concomitant medications are shown in Table 2. Among the included patients, all had evaluable data for 3 months of follow-up; 13 (6.5%) individuals were lost by the 6-month follow-up. Due to crossover between the TRA and TUA groups, TRA was finally used in 120 (60%) patients, TUA in 77 (38%) patients, and the transbrachial artery (TBA) in 3 (2%) patients. QC was used in 100 patients (TRA: 60 patients; TUA: 39 patients; TBA: 1 patient) and SC in 100 patients (TRA: 60 patients; TUA: 38 patients, TBA: 2 patients). A standard bolus of UFH 5000 IU was administered during the diagnostic CAG. During PCI, additional UFH was administered intravenously to reach a 1 mg/kg dose. The median dose of UFH in both groups was similar (QC: 5000 ± 1626 UI; SC: 5000 ± 1772 UI [*p* = 0.8]). Before CAG, aspirin and clopidogrel were routinely administered to the patients as preparation for eventual PCI (Table 1).

**Table 1:** Patients’ baseline characteristics and concomitant medications in the QC and SC groups

Parameter	QC (*n* = 100)	SC (*n* = 100)	*p*
Male sex, *n* (%)	49 (49)	42 (42)	0.32
Age in years, mean ± SD	64 ± 9.1	67 ± 10	0.01
Hypertension, *n* (%)	81 (81)	81 (81)	1.00
Hypercholesterolemia, *n* (%)	71 (71)	76 (76)	0.42
Peripheral artery disease, *n* (%)	13 (13)	6 (6)	0.09
Diabetes, *n* (%)	33 (33)	29 (29)	0.54
Stroke, *n* (%)	6 (6)	6 (6)	1.00
Renal insufficiency, *n* (%)	2 (2)	5 (5)	0.25
Medication			
Aspirin, *n* (%)	99 (99)	98 (98)	0.67
Clopidogrel, *n* (%)	97 (97)	97 (97)	0.28
Statin, *n *(%)	61 (61)	63 (63)	0.77
β-blocker, *n* (%)	76 (76)	76 (76)	1.00
ACE-I/ARB, *n* (%)	66 (66)	66 (66)	1.00
Ca-channel blocker, *n* (%)	29 (29)	31 (31)	0.75
Warfarin, *n* (%)	7 (7)	7 (7)	1.00
Novel oral anticoagulant, *n* (%)	3 (3)	6 (6)	0.30

*QC* QuikClot Radial hemostatic pad, *SC* standard, manual compression system

**Table 2:** Periprocedural data

	QC (*n* = 100)	SC (*n* = 100)	*p*
Angiography alone, *n* (%)	64 (64)	63 (63)	0.88
Ad hoc PCI, *n* (%)	36 (36)	36 (36)	1.00
Arterial sheath size			
6-Fr	100	100	1.00
Diagnostic catheter size			
6-Fr, *n* (%)	93 (93)	88 (88)	0.2

	PCI (*n* = 36)	PCI (*n* = 36)	*p*
Catheter sizes used for PCI			
6-Fr, *n *(%)	36 (36)	35 (97)	0.9

*PCI* percutaneous coronary intervention, *Fr* French catheter scale

Diagnostic CAG was performed in all patients (*n *= 200), while ad hoc PCI was conducted in 36 (36%) and 36 (36%) patients from the QC and SC groups, respectively. In 7 (7%) patients of the QC group and in 12 (12%) patients of the SC group, 5-Fr diagnostic catheters were used. Angiographic and procedural data were similar between the two groups (Table 2).

**Fig. 3:**
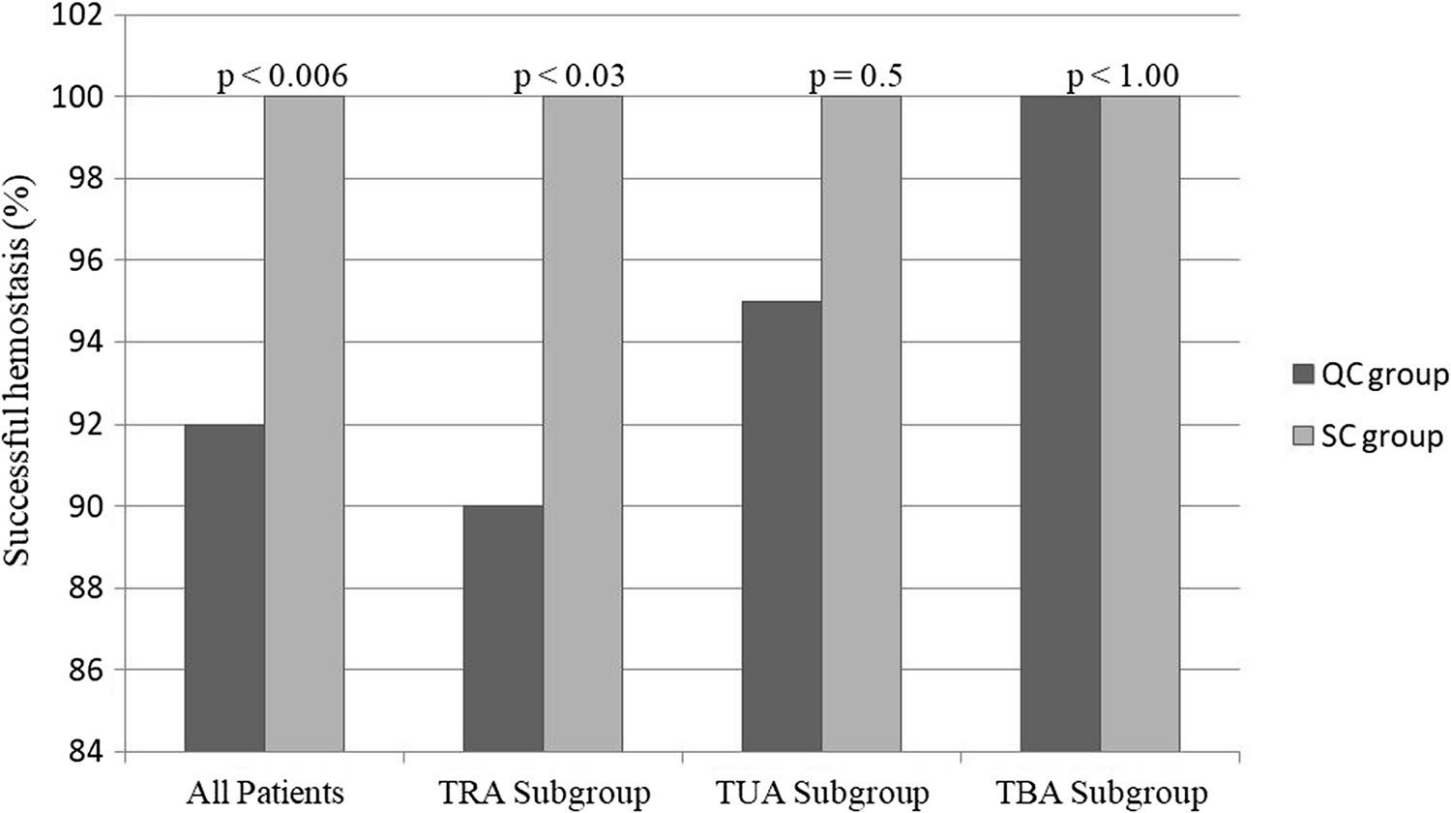
Efficacy of hemostasis in the QC and SC groups and the TUA and TRA subgroups

A statistically significant difference (*p* < 0.0065) was found in the distance from the forearm arteries to the skin only for the QC group (Table 3). Other comparisons (diameter, PSV) showed no significant differences. Primary endpoint (successful hemostasis) was achieved in 92 (92%) patients in the QC group and in 100 (100%) patients in the SC group (*p* < 0.006) (Table 4). In the QC group, most bleeding events occurred after removal the QC pad (7 patients) versus during compression in only one patient. In the subgroup analysis of hemostasis success, TRA QC showed significantly better results than TRA SC (54 [90%] patients vs. 60 [100%] patients; *p* < 0.03), while similar results were obtained in the TUA QC and TUA SC subgroups (37 [95%] patients vs. 38 [100%] patients; *p* = 0.5) (Fig. 3). In the TBA subgroup (QC: 1; SC: 2), hemostasis was also achieved in all individuals. No statistically significant differences in safety were found between the QC and SC groups. The frequency of composite endpoint (TAO, IPA, large hematoma) was similar in the QC and SC groups (8% vs. 9%; *p* = 0.8). After the 3-month follow-up, TAO occurred in four patients in the QC group and three patients in the SC group. We noticed that the number of TAO events increased over time in both groups. An IPA was diagnosed in 2 (2%) cases in both groups. Large hematomas (grade 4 on the EASY scale) occurred in 5 (5%) patients in the QC group and in 3 (3%) patients in the SC group. The most common complications were small hematomas (grades 1–3 on the EASY scale) in both groups. There were also no statistically significant differences in safety between the TRA and TUA subgroups. An analysis of VAS data revealed that patients reported significantly less pain during the assumed QC vs SC regardless of the access (2.6 ± 2.6 vs. 3.4 ± 2.9; *p* < 0.03). All patients from both groups were discharged at a mean 1.95 ± 0.3 days.

**Table 3:** Anatomical US data of RA and UA

	QC	SC	*p*
RA (*N* = 120)	(*n* = 60)	(*n* = 60)	
Distance: skin to RA (mm), mean ± SD	5.64 ± 2.26	5.56 ± 2.22	0.85
UA (*n* = 78)	(*n *= 39)	(*n* = 38)	
Distance: skin to UA (mm), mean ± SD	7.05 ± 2.7821	6.3 ± 2.3	0.19
*p*	0.0065	0.12	

*US* ultrasonography, *RA* radial artery, *UA* ulnar artery

**Table 4:** Efficacy of hemostasis in the QC and SC groups and the TUA and TRA subgroups

Successful hemostasis	QC (*n* = 100)	SC (*n* = 100)	*p*
All groups, *n* (%)	92 (92)	100 (100)	0.006
	QC (*n* = 60)	SC (*n* = 60)	
TRA subgroup, *n* (%)	54 (90)	60 (100)	0.03
	QC (*n* = 39)	SC (*n* = 38)	
TUA subgroup, *n* (%)	37 (95)	38 (100)	0.5
	QC (*n* = 1)	SC (*n* = 2)	
TBA subgroup, *n* (%)	1 (100)	2 (100)	1.00

QC QuickClot Radial compression pad, *SC* standard manual compression, *TRA* transradial access, *TUA* transulnar access, *TBA* transbrachial access

## Discussion

The main finding of this study was that QC compression system showed lower hemostasis efficacy than SC in forearm vascular access. Both hemostasis methods showed high safety levels. Analysis of the VAS data revealed that QC was associated with significantly less pain than SC regardless of the access. The efficacy of QC seemed to be comparable to that of SC in the TUA subgroup; however, the number of patients of both subgroups was too small to formulate such conclusion. TRA, the main vascular access in invasive cardiology, was confirmed as a safer approach than transfemoral access (TFA) access in many trials. TRA was found to reduce the frequency of bleeding and mortality after CAG in ACS [17, 18]. Many compression devices dedicated to TRA or TFA are currently available on the market and are used to improve patient comfort and reduce the frequency of variety complications and compression time [3, 13].

New trials concerning QC Radial have been conducted. One of the newest trials assessed the time of stable cloth formation and efficacy of hemostatic device in the QC Radial and TR band groups (Roberts J et al.) [19]. However, many cath labs still use simple manual SC due to low cost and high hemostasis efficiency. The use of UA as vascular access has been increasing systematically. Many trials confirmed efficacy and safety of this vascular access [20–23]. TRA and TUA applied in a group of 1100 patients scheduled for CAG reduced the need for conversion TFA to less than 1% of cases [24]. However, no dedicated hemostatic systems are currently available for this artery. The UA has a specific anatomy, larger diameter, straighter course, lower predisposition to spasm, and deeper location than RA. This last feature may be one of the main reasons for the higher rates of hemostasis failure, bleeding complications, and puncture failure compared to those with TRA [22]. The distance from the skin to the artery may be one of the few influencing factors of the efficacy of QC. It is possible that the use of QC and its double mechanism of hemostasis (compression and clot activation) may be more efficient for the TUA than for the TRA because of the deeper location of the UA. In that vascular access, the thrombus seems longer, larger, and more stable than TRA. The QC hemostatic device is known to be efficient and safe when CAG is performed with deep located FA. Trabattoni et al. assessed two methods of hemostasis after CAG with TFA, kaolin-based hemostatic bandage and manual compression. The time to achieve successful hemostasis was significantly shorter in the kaolin-based hemostatic bandage group than in the manual compression group (5.4 ± 1.5 min vs. 25 ± 15 min, *p* < 0.001), but efficacy was similar in both groups [11]. In another study, Politi et al. defined factors that influenced the efficacy of QC, including chronic therapy with clopidogrel (odds ratio [OR] 28.78; 95% confidence interval 4.79–172.92; *p* < 0.001) and long activated clotting time (ACT) at the time of sheath removal (OR 1.02; 95% CI 1.00–1.03; *p* = 0.009). The ACT associated with the risk of bleeding was 287 s, with a sensitivity of 80% and specificity of 75% [12]. With the QC hemostatic device, the time of thrombus formation, thrombus stability, and resistance for lysis were greater than those in thrombus created by standard processes of hemostasis using SC. These data were presented in a small study by Lechner et al. The aim of this trial was to assess the efficacy of hemostatic dressings in human blood [25]. Protocols of using QC in real life have differences depending on local experiences. Protocols in main trials also differ concerning this hemostatic device. In a pilot trial that compared QC and the TR band, Roberts et al. tested 30 and 60 min of full compression of QC, leaving the QC roll till morning (no evidence was found that the QC roll was left up to 24 h) [13]. In another trial, Chiang et al. used QC differently from the usage recommended by the manufacturer, and the time of QC removal was not exactly defined. RAO risk was not significantly different between the QC and control groups after 24  h (4.6% vs. 5.4%, *p* = 0.776) or after 1 month (5.4% vs. 6.1%, *p* = 0.789) [26]. In the trial of Politi et al., the QC roll was applied for 15 min of full compression and then for 2 h of slight compression (group 1). In the second group, SC was applied only for 15 min, and in the third group, SC was applied for 2 h. The main outcome of this study was significant reduction of RAO in the QC group (none of the patients in group 1 developed RAO, 1 [5%] occurred in group 2, and 5 [10%] occurred in group 3; *p* = 0.05). Active bleeding (efficacy) after compression removal occurred in ten patients (20%) in group 1, 18 (90%) in group 2, and 1 (2%) in group 3 (*p* < 0.001) [12]. The optimal time of slight compression with the QC roll is unknown and may have an impact on the efficacy and safety of this hemostatic device. It should also be noted that the use of QC is more comfortable regardless of the approach. This may be important in the TUA group, in which compression may be associated with greater discomfort related to the need to apply higher pressure to achieve hemostasis. This fact could be crucial to avoid forearm disorders after CAG or PCI [2, 7]. The incidence of other complications, including IPA or TAO, did not differ from the frequency reported in previous publications (Table 5) [2, 27, 28]. The use of QC and SC in patients was also not associated with prolonged hospitalization regardless of the vascular approach. The increase in the number of arterial occlusions during the observation period was observed. The number of TAO in the entire study group tripled at the 6-month follow-up. This observation requires further research.

**Table 5:** Frequency of complications and VAS of compression pain during hemostasis

Complication	QC (*n* = 100)	SC (*n* = 100)	*p*
Large hematoma, *n* (%)	5 (5)	3 (3)	0.72
IPA, *n* (%)	2 (2)	2 (3)	1.00
VAS compression (points 0–10)	2.6 ± 2.6	3.4 ± 2.9	0.03
TAO at 1-month follow-up, *n* (%)	0	2 (2)	0.5
TAO at 3-month follow-up, *n* (%)	2 (2)	2 (2)	1.00
	QC (*n* = 94)	SC (*n* = 93)	
TAO 6-month follow-up, *n* (%)	4 (4)	3 (3)	0.9

*TAO* total artery occlusion, *IPA* iatrogenic pseudoaneurysm, *VAS* visual analog scale

## Study limitations

The QC Radial pad was designed for TRA; its utility for TUA was unknown and not previously investigated. Thus, instructions for its use with this approach were unavailable. Second, the compression time may have been too long in both groups, possibly affecting the TAO. Third, the QC Radial roll could be applied with slight compression for more than 120 min, possibly improving efficacy in the TRA subgroup. We did not measure the ACT level before removal of the vascular sheath. High ACT level could be one of the reasons of the failure of hemostatic devices. We did not perform a Barbeau test but did assess circulation in the deep and superficial arch using Doppler ultrasonography. Finally, 13 patients (6.5%) were lost to long-term follow-up and the actual number with TAO after 6 months could be biased.

## Conclusions

This trial compared the QC hemostatic system with SC with TRA and TUA and found that the efficacy of QC was significantly lower than that of SC. Safety was similar between the QC and SC groups. QC improved patient comfort regardless of the access site. However, QC efficacy seemed to be comparable to that of SC in the TUA subgroup. This requires further studies. Because of anatomical differences between RA and UA, development of a dedicated hemostatic device for TUA is recommended.
